# Skin Reactions in Children with Type 1 Diabetes Associated with the Use of New Diabetes Technologies—An Observational Study from a Regional Polish Pediatric Diabetes Center

**DOI:** 10.3390/children11060740

**Published:** 2024-06-17

**Authors:** Ewa Ledwoń, Paula Zemła-Szten, Thekla von dem Berge, Krzysztof Nalewajko, Stefano Passanisi, Claudia Piona, Tiago Jeronimo dos Santos, Jannet Svensson, Anna Korsgaard Berg, Agata Chobot

**Affiliations:** 1 Department of Pediatrics, University Clinical Hospital in Opole, 45-040 Opole, Poland; ewa.ledwon@uni.opole.pl (E.L.); agata.chobot@uni.opole.pl (A.C.); 2 Faculty of Health Sciences, University of Opole, 45-060 Opole, Poland; 3 Department of Pediatrics, Institute of Medical Sciences, University of Opole, 47-100 Strzelce Opolskie, Poland; 4 Auf der Bult, Diabetes Center for Children and Adolescents, 30173 Hannover, Germany; vondemberge@hka.de; 5 Department of Cardiology, Institute of Medical Sciences, University of Opole, 45-040 Opole, Poland; krzysztof.nalewajko@uni.opole.pl; 6 Pediatric Unit, Department of Human Pathology in Adult and Developmental Age “Gaetano Barresi”, University of Messina, 98122 Messina, Italy; stefano.passanisi@unime.it; 7 Department of Surgery, Dentistry, Gynecology and Pediatrics, Section of Pediatric Diabetes and Metabolism, University and Azienda Ospedaliera Università di Verona, 37126 Verona, Italy; claudia.piona@univr.it; 8 Pediatrics Unit, Hospital Vithas Almería, Instituto Hispalense de Pediatría, 04120 Almería, Spain; tjdossantos@ual.es; 9 Department of Nursing Sciences, Physiotherapy, and Medicine, Faculty of Health Sciences, University of Almería, 04120 Almería, Spain; 10 Steno Diabetes Center Copenhagen, 2730 Herlev, Denmark; jannet.svensson.01@regionh.dk (J.S.); anna.korsgaard.berg@regionh.dk (A.K.B.); 11 Department of Clinical Medicine, University of Copenhagen, 2200 Copenhagen, Denmark; 12 Department of Biomedical and Clinical Sciences, Linköping University, 58183 Linköping, Sweden

**Keywords:** type 1 diabetes, children, skin problems, insulin pump, CGM

## Abstract

The study aimed to estimate the prevalence of skin problems in children and adolescents with type 1 diabetes (T1D) using insulin pumps (IPs) and/or continuous glucose monitoring (CGM) in our center and analyze their association with various factors. As part of the international ISPAD JENIOUS-initiated SKIN-PEDIC project, we interviewed and examined patients who visited the regional pediatric diabetes center in Opole (Poland) for four weeks regarding the use of IP and/or CGM and the presence of skin problems. Body mass index (BMI) and glycemic parameters were obtained retrospectively from medical records. Among 115 individuals (45.2% girls, 83.5% IP users, 96.5% CGM users), old scars were the most common skin problem (IP users 53.1%; CGM users 66.4%), while ≥2 types of skin problems co-occurred (IP users 40.6%; CGM users 27.3%). Longer IP use was associated with a higher prevalence of skin problems (50% for IP < 1 year, 98.1%-IP 1–3 years, 100% for IP > 3 years; *p* < 0.001), pointing out extra attention with IP use > 1 year. No significant associations were found between skin problems and gender, age, BMI centile and glycemic parameters. Dermatological complications were common among children using IP and CGM in our center, highlighting the need for vigilant monitoring and early intervention to manage these skin-related issues effectively.

## 1. Introduction

The use of modern technologies, such as insulin pumps (IPs) and continuous glucose monitoring (CGM) systems, in the management of type 1 diabetes (T1D) in children and adolescents is continuously increasing [1,2,3]. Their use improves glycemic control, reducing the risk of long-term vascular complications and life-threatening acute complications of T1D [1,2,4]. The positive impact on patients’ quality of life is well documented [5,6]. It needs to be acknowledged that regardless of the undoubted benefits of using new diabetes technologies, possible complications may hinder their use, leading in extreme cases to discontinuing IPs or CGM. Therefore, increasing attention is drawn to dermatological complications [7].

Most CGM devices are registered for use for 7–14 days, while insulin infusion sets (IISs) must be replaced every 2–7 days [8]. Additional patches and adhesive tapes are often recommended and used to ensure adhesion to the skin [8,9]. In places where IP or CGM are inserted, itching, wounds, scars, infections, allergic or lipodystrophic changes [10], and other lesions may appear. Sometimes, skin problems develop due to disinfectants or skin protection agents [11,12]. A review of publications from 2018 to 2019 found that allergic contact dermatitis (ACD) is common in individuals using modern technologies, including people living with diabetes. Unfortunately, manufacturers are often not willing to provide the full composition of their products. According to this review, dermatologists in cooperation with chemists determined that isobornyl acrylate or N,N′-dimethylacrylamide may be related to cases of ACD as a reaction to CGM or IP [13]. Also, many children use additional adhesives or materials as well as different kinds of skin preparations. The variety of these and often the lack of a complete list of their chemical compounds may hinder the clinical care as well as research on this topic. It is, therefore, crucial to increase the knowledge of skin problems related to diabetes devices, optimally treat possible dermatological complications and find solutions to prevent them. The study aimed to estimate the frequency of skin problems in children with T1D under the care of our regional pediatric diabetes center and analyze potential associations of the occurrence of skin problems with various parameters, including anthropometric and glycemic control parameters.

## 2. Materials and Methods

The study was part of the SKIN-PEDIC research project (Bioethics Committee of the University of Copenhagen approval no. P-2022-531)—an international collaboration of the International Societies for Pediatric and Adolescents (ISPAD) JENIOUS group. For four weeks, between January and February 2023, we enrolled all children and adolescents with T1D aged from 2 to 18 years old using diabetes devices (either IP, CGM or both) and visiting the Department of Pediatrics and Pediatric Diabetes Outpatient Clinic at the University Clinical Hospital in Opole participating in SWEET as center of reference.

Children and their caregivers in our center are educated (from T1D onset) and periodically, at least every 3 months, re-educated about skincare and skin problems according to the current position statement of the Polish Federation for Education in Diabetology [14]. The principles of IIS (insulin infusion set) and CGM sensor placement and care for the insertion sites are discussed in detail, and this knowledge is verified. We ask to change the site in a “6-times cycle” (each side of the abdomen, buttocks, and thighs) for insulin administration every 3 days. When returning to a site during this cycle, the area should be moved at least by 6 cm. Children and youth under the care of our center may use all IP and CGM models available on the Polish market. Individuals participating in the study used most of the available at that time IISs (MiniMed™ Quick-set™, Silhouette^TM^, Sure-T^TM^, Medtronic Extended, MiniMed Mio 30°, MiniMed Mio Advance, Orbit micro, Orbit soft, Accu Chek Tender Link, Accu Chek FlexLink) and glucose sensors (Enlite CGM-RT, Guardian^TM^ Sensor 3, Guardian^TM^ Sensor 4, FreeStyle Libre 2, Dexcom G6).

For each participant, following the SKIN-PEDIC study Phase 2 protocol, the following data were collected at one data point: age (age groups: <4 years, 4 to <8 years, 8 to <13 years, 13 to 18 years), gender, method of insulin therapy, type of IP and CGM and the duration of their use (<6 months, 6 months to <1 year, 1 year to 3 years, >3 years), type of insulin administered, other products used to insert and remove the glucose sensor and IIS and the presence of other dermatological problems (e.g., dry skin or keratosis pillars). In each individual, we assessed the skin type according to Fitzpatrick [15] and the skin condition at the site of device insertion for skin problems categorized into the following: wounds, old scars, lipoatrophy, lipohypertrophy, eczema, changes suggesting infection, or other reactions.

Additionally, anthropometric data and glycemic control parameters (HbA1c (%), time spent within the glucose range of 70–180 mg/dL (TIR, %), coefficient of variation (CV, %) from the previous 14 days were obtained retrospectively from the medical records from the visit during which the skin was assessed as part of the SKIN-PEDIC study. Body mass index (BMI) was calculated and presented as percentiles (for age and sex, using the respective growth charts for the Polish population [16]).

The analysis was performed using R software version 4.2.3. Descriptive data are presented as mean ± standard deviation (SD), median, range and percentage when appropriate. To compare the means of different groups, we used the ANOVA parametric test, which assumes normality and homogeneity of variance, and the Kruskal–Wallis non-parametric test, which does not require these assumptions. We chose a significance level of 0.05 for both tests.

## 3. Results

Of the 119 children and adolescents that participated in the study (all seen in the center and eligible by the above-mentioned inclusion criteria), four were excluded from the analysis due to incomplete data regarding skin problems. The studied group included therefore 115 children (52 (45.2%) girls and 63 (54.8%) boys). Ninety-six (83.5%) used an IP, 110 (96.5%) used a CGM, and 91 (79.1%) used both—IP and CGM. Seventy-two individuals (62.6%) presented dry skin, and 52 (44.1%) had keratosis pilaris. According to Fitzpatrick, most children had skin type II, and only 2 (1.7%) had skin type I. The characteristics of the group are presented in Table 1.

Of the children using both IP and CGM systems, only five children (5%) had no skin complications. Skin problems were not observed in seven children at the CGM sensor site (6.4% of users) and in six children at the IIS sites (6.3% of users). The most common issue was the presence of old scars at the sites of IIS and sensor insertion, occurring in 53.1% (51 out of 96) and 66.4% (73 out of 110) of the children, respectively. Additionally, lipohypertrophy was identified in 20.9% of the participants overall with 18.8% at the IISs sites and 10.9% at the CGM sensor sites. At the IISs sites, ≥2 types of concomitantly skin problems were present in 39 out of 96 (40.6%) children, which were mainly old scars with lipohypertrophy. Only one child had solely lipohypertrophic lesions. In the sensor area, ≥2 types of concomitant lesions were present in 30 out of 110 (27.3%) children—mainly old scars and wounds or old scars with lipohypertrophy. None of the study participants reported having to stop using IP/CGM due to skin problems.

No significant differences in the frequency of skin problems depending on gender and age group were found (Table 2). Skin problems occurred significantly more often in children with dry skin (*p* = 0.017 for IIS and *p* = 0.023 for CGM) (details in Table 2).

In the majority, IP and CGM were used for more than 1 year at the time of the study (86/96, 89.6% and 86/110, 81.1%, respectively). Skin problems at the IIS site occurred in half of the respondents (5/10, 50%) using the IP for less than a year, in almost all (52/53, 98.1%) using the IP for 1–3 years (mainly old scars, 60.4%) and in all IP users > 3 years (33/33, 100%; mainly only old scars (19/33, 57.6%) or ≥2 types of changes (14/33, 42.4%). Although skin problems were observed more commonly with an increasing duration of IP use (*p* > 0.001), there were no differences in their frequency between groups with different CGM use duration (*p* = 0.052).

We also found a varying prevalence of skin problems depending on the products participants use when inserting the IIS or sensor (for both *p* < 0.001). Old scars at the CGM site were more common in those using disinfection spray or wipe before inserting the sensor than those that did not (59/75, 78.7% vs. 2/7, 28.6%, *p* = 0.004). We did not observe more frequent old scars at the IIS site regarding this kind of disinfection (39/59, 66.1% vs. 4/9, 44.4%, *p* = 0.209). Children disinfecting with fluids or wipes before placing the IIS (59/95, 62.1%) or sensor (75/110, 68.2%) mainly had old scars (respectively, 39/59, 66.1% and 59/75, 78.7%). Slightly fewer children (IP: 21/95, 22.1% and CGM: 23/110, 20.9%) reported using ≥2 different agents simultaneously before placing the IIS or CGM (disinfectant wipes and liquids, additional patches/patches to increase adhesion, spray or liquid barrier wipes). From this subgroup, all had skin problems at the IIS site and 14/23 (60.9%) had problems at the CGM site, mainly ≥2 types of skin problems. Nine children declared that they did not use any preparations for their IIS placement, and none used intranasal steroids when inserting the sensor or IIS.

Differences in the frequency of skin problems among insulins were noted (*p* = 0.017). Children using NovoRapid^®^ or Humalog^®^ had predominantly only isolated old scars (respectively, 18/29, 62.1% and 30/50, 60.0%) and less often ≥2 types of skin problems (respectively, 10/29, 34.5% and 16/50, 32%). In children using Fiasp^®^, most often ≥2 types of skin problems (13/17, 76.5%) were observed.

The mean values of the analyzed glycemic control parameters among the investigated children and adolescents were HbA1c—7.62 ± 1.23%, TIR—64.0 ± 17.5%, and CV—41.9 ± 12.5%. We found no significant differences in these parameters and BMI centile between groups of patients with different types of skin problems (Table 3).

## 4. Discussion

Most children and adolescents with T1D from our diabetes center who use diabetes technologies face skin problems in daily life. Similar observations pointing to the problem of dermatological complications related to the use of modern technologies also come from previous studies [11,17,18]. Among our subjects, as in other reports, the predominant lesions were old scars [18,19,20], which may result from chronic or repeated skin damage. Among the study participants, lipohypertrophy was demonstrated at the site of the IIS insertion and the sensor site. Since this change is primarily the result of repeated insulin administration in one place, leading to the local hypertrophy of adipose tissue [21,22], it may suggest that children chose these places to place sensors—maybe because of the convenience of access or due to less pain. Although insulin absorption from areas affected by lipohypertrophy is impaired [9,22], one study showed that inserting a CGM sensor in such a place did not affect the accuracy of the readings compared to the sensor on healthy skin [22]. However, another study demonstrated the impact of lipodystrophy on glycemic variability metrics [23].

We did not demonstrate any association between the frequency of skin problems and the values of glycemic control parameters—HbA1c, TIR and CV. Similarly, no relation with HbA1c levels has been observed in previous studies [7,17,18,23]. Additionally, the Italian study that revealed a negative impact of lipodystrophy on CV (even after adjusting for age) also described that other CGM metrics (TIR, time spent in the range >180 mg/dL, time spent in the range < 70 mg/dL, and mean blood glucose) were not influenced by the presence of lipodystrophy [23]. Although our analysis and some of the other studies included all types of skin problems [7,17,18], it seems that these might develop regardless of the individual’s metabolic outcomes, and skincare is critical in every case. However, the impact of specific skin problems may depend on their type and should be further investigated. Additionally, while obesity is a factor that increases the risk of infection and causes poorer wound healing [24], we did not find higher BMI or body weight values in children with skin problems. Mean BMI centiles were within the normal range for all studied groups as well as in subgroups.

The differences in the frequency of skin problems among insulins need to be interpreted with caution, as such findings have not been reported previously.

The scale of occurrence of skin problems and their appearance in quite a large group of children, even in the first year of using new diabetes technologies, emphasizes the importance of educating people with diabetes and their families in this area. Although scars are challenging to prevent, perhaps longer healing time and omitting sun exposure may have a positive effect. For preventing eczema and wounds, a correct technique of installing devices, timely replacement, appropriate skin care before placing sensors or IIS, and individual equipment adjustment are important. The development of lipodystrophy can be decreased, for example, by site rotation. It is also vital to perform a thorough physical examination with skin assessment during follow-up visits and to follow the recommendations to prevent or quickly treat existing lesions [8,9,25]. The most important recommendations based on the literature are listed in Table 4.

It seems that one of the critical elements of preventing skin problems is understanding the conditions related to the use of new technologies (e.g., regular replacement of IIS and sensors) and early handling of the skin problems. In a Finnish study, people with type 1 diabetes had suspected allergic contact dermatitis caused by CGM sensors. All study participants had mild/severe contact dermatitis. The performed patch tests were positive for isobornyl acrylate (81% of Freestyle Libre users) or to both, colphonium and the sensor’s adhesive overtape (57% Enlite users). Some of these individuals were not able to continue using the sensor or changed sensor type [27]. Another study, published in 2015, showed no significant association between the material from which the IIS or the cannulas of the IIS were made (steel/teflon) and the prevalence of skin reactions [18].

If access to different devices is available, the IP, the type of catheter, and the CGM system should be adapted to the individual needs of the person with diabetes. This also applies to adhesives and additional protective patches that prepare the skin for the injection/sensor insertion as well as oils and preparations for removing the remaining glue. Whereas cleaning and disinfecting the injection site using available agents (they may or may not contain alcohol), e.g., wipes and liquids, are advised by most recommendations, it may predispose to the more frequent development of scars. Therefore, disinfectants should be omitted instead [12]. In our study, the types of IISs, CGM systems, and adhesive removers did not show a significant association, but these calculations were underpowered.

The study’s limitations were primarily the cross-sectional design and retrospective acquisition of some data, including those regarding glycemic control. This resulted in limited access to complete CGM readings and only allowed obtaining information contained in the medical records. Due to the above, sufficiently precise information was not obtained regarding the frequency of replacing the sensor and IISs, which would have allowed for a reliable assessment of this variable. Another limitation for associations of different variables to having skin problems is the low power of individuals not suffering from skin problems. A future investigation would benefit from a prospective design and adding a control group. The homogeneity of the ethnic background of the study participants did not allow for evaluation of the potential influence of different skin types on the occurrence of skin problems. The study’s strength is the protocol-compliant and systematic acquisition of information about skin problems in an unselected and representative sample of all children reporting to a diabetes center within a specified period. Moreover, an original element of the presented study is the analysis of the frequency of skin problems concerning not only HbA1c but also CGM parameters.

## 5. Conclusions

Dermatological complications are a significant problem among children and adolescents with T1D using modern diabetes technologies under the care of a diabetes center in Poland. Skin problems can manifest already within the first year of using the devices. Such issues were observed in every participant using a diabetes device for over 3 years. The frequency of skin problems was not associated with HbA1c, CGM metrics or anthropometric measurements. The widespread use of modern technologies in the pediatric age group requires efforts to decrease this problem by repeated diabetes education emphasizing recommended skincare, verifying the individuals’ practices and developments regarding products that have direct contact with the skin (patches, adhesives and other substances).

## Figures and Tables

**Table 1:** Characteristics of the whole studied group and subgroups using an insulin pump (IP) or continuous glucose monitoring system (CGM). The differences between age groups and gender for the whole group, IP and CGM users were not significant.

		TOTAL (n = 115)	IP (n = 96)	CGM (n = 110)
Age	<4 years	3 (2.6%)	3 (3.1%)	3 (2.7%)
	4–7 years	13 (11.3%)	13 (13.5%)	13 (11.8%)
	8–12 years	50 (43.5%)	44 (45.8%)	48 (43.6%)
	13–18 years	49 (42.6%)	36 (37.5%)	46 (41.8%)
Sex	Male	63 (54.8%)	50 (52.1%)	61 (55.5%)
	Female	52 (45.2%)	46 (47.9%)	49 (44.5%)
Time of IP use	<6 months	2 (2.1%)	2 (2.1%)	2 (2.2%)
	6–12 months	8 (8.3%)	8 (8.3%)	8 (8.8%)
	1–3 years	53 (55.2%)	53 (55.2%)	53 (58.2%)
	>3 years	33 (34.4%)	33 (34.4%)	28 (30.8%)
Time of CGM use	<6 months	1 (0.9%)	1 (1.1%)	1 (0.9%)
	6–12 months	19 (17.9%)	17 (19.5%)	19 (17.9%)
	1–3 years	63 (59.4%)	52 (59.8%)	63 (59.4%)
	>3 years	23 (21.7%)	17 (19.5%)	23 (21.7%)
Dry skin		72 (62.6%)	58 (60.4%)	70 (63.6%)
Keratosis piliaris		49 (43.0%)	37 (38.9%)	48 (43.6%)
HbA1c (%)		7.62 ± 1.23	7.55 ± 1.22	7.55 ± 1.08
BMI (centile)		61.2 ± 26.6	60.5 ± 25.3	60.9 ± 26.6
TIR (time in range 70–180 mg/dL)		64.0 ± 17.5	65.0 ± 17.9	64.0 ± 17.1
CV (%)		41.9 ± 12.5	40.7 ± 12.0	41.7 ± 12.0

HbA1c—glycated hemoglobin A1c, BMI—body mass index, TIR—time in range, CV—coefficient of variation.

**Table 2:** Frequency of skin problems (n (%)) in the area of insertion of the insulin pump (IP) infusion set or continuous glucose monitoring (CGM) sensor.

		Insulin Infusion Set Site				CGM Sensor Placement Area			
		No skin problems	Isolated old scars	≥2 types of skin problems	*p*	No skin problems	Isolated old scars	≥2 types of skin problems	*p*
Sex	Male	4 (8.0%)	24 (48.0%)	22 (44.0%)	NS	5 (8.2%)	43 (70.5%)	13 (21.3%)	NS
	Female	2 (4.3%)	27 (58.7%)	17 (37.0%)		2 (4.1%)	30 (61.2%)	17 (34.7%)	
Age group	<4 years	1 (33.3%)	0 (0%)	2(66.7%)	NS	1 (33.3%)	1 (33.3%)	1 (33.3%)	NS
	4–7 years	1 (7.7%)	7 (53.8%)	5 (38.5%)		2 (15.4%)	7 (53.8%)	4 (30.8%)	
	8–12 years	2 (4.5%)	27 (61.4%)	15 (34.1%)		2 (4.2%)	35 (72.9%)	11 (22.9%)	
	13–18 years	2 (4.5%)	17 (47.2%)	17 (47.2%)		2 (4.3%)	30 (65.2%)	14 (30.4%)	
Dry skin	no	4 (10.5%)	25 (65.8%)	9 (23.7%)	0.017	4 (10.0%)	33 (77.5%)	5 (12.5%)	0.023
	yes	2 (3.4%)	26 (44.8%)	30 (51.7%)		3 (4.3%)	25 (35.7%)	25 (35.7%)	
Duration of device use (IP or CGM, respectively)	<6 months	1 (50.0%)	0 (0%)	1 (50.0%)	<0.001	0 (0%)	0 (0%)	1 (100%)	NS
	6–12 months	4 (50.0%)	0 (0%)	4 (50.0%)		4 (21.1%)	9 (47.4%)	6 (31.6%)	
	1–3 years	1 (1.9%)	32 (60.4%)	20 (37.7%)		3 (4.8%)	43 (68.3%)	17 (27.0%)	
	>3 years	0 (0%)	19 (57.6%)	14 (42.4%)		0 (0%)	18 (78.3%)	5 (21.7%)	

NS—not significant.

**Table 3:** Glycemic control parameters (TIR—time in range 70–180 mg/dL. CV—coefficient of variation. HbA1c—glycated hemoglobin) and body mass index (BMI) in children and adolescents with type 1 diabetes using an insulin pump (IP) or continuous glucose monitoring system (CGM) regarding the presence or absence of specific skin problems (ANOVA for all parameters for both IP and CGM *p* > 0.05).

	IP/CGM	No Skin Problems	Isolated Old Scars	Two or More Types of Skin Problems
HbA1c (%)	IP	7.23 ± 1.14	7.58 ± 1.43	7.55 ± 0.91
	CGM	7.59 ± 1.38	7.54 ± 1.08	7.57 ± 1.04
TIR (%)	IP	69.7 ± 18.5	64.3 ± 18.9	65.2 ± 16.8
	CGM	64.1 ± 21.4	63.2 ± 17.6	66.2 ± 14.8
CV (%)	IP	39.2 ± 11.5	41.1 ± 12.2	40.3 ± 11.9
	CGM	39.4 ± 9.6	42.4 ± 12.5	40.6 ± 11.7
BMI (centile)	IP	69.3 ± 23.1	59.5 ± 22.7	60.5 ± 28.9
	CGM	64.0 ± 20.4	59.0 ± 26.9	64.7 ± 27.3

**Table 4:** Recommendations for skincare concerning the use of diabetes technologies—insulin pump (IP) infusion set placement and continuous glucose monitoring (CGM) sensor insertion; developed based on [9,12,14,26].

○ Selecting the appropriate place to place the CGM sensor according to the label: - Upper part of the buttocks; - Belly (avoid places that tend to create folds. preferably about 2.5 cm from the navel); - Upper part of the thigh; - Arm. ○ Assessment of the injection site—avoiding irritated skin with signs of damage. ○ Whereas most recommend cleaning and disinfecting the injection site using available agents (they may or may not contain alcohol), e.g., wipes, liquids, it may predispose patients to a more frequent development of scars [12]. When a disinfectant is used anyway, the skin should be left to dry (time specified by the disinfectant manufacturer). ○ Additional skin preparation: - Trimming the hair in the planned place where the set will be placed; - In case of excessive sweating, you can apply antiperspirant to the skin 10–15 min before planning to put on the set. ○ To reduce the risk of allergic reactions, it is allowed to use barrier foil or patches under the sensor tape. ○ Preparation of the sensor. Serter (device for installing the sensor) and mounting patch. Remember that the sensor should be applied in a dry environment—do not put it on immediately after a bath or shower. ○ Use of additional security systems, e.g., tapes, adhesives. ○ Following the manufacturer’s recommendations regarding the service life of cannulas and infusion sets. ○ Replacing the infusion set if the following apply: - Symptoms of infection at the injection site (redness. swelling. pain. itching); - Presence of blood in the drain; - Increase in glycemia despite proper insulin dosing. ○ Tapes and adhesives should be removed carefully; additional preparations may be used. ○ After removing the sensor, it is necessary to clean the skin of any adhesive residue. ○ Regularly changing the injection site to maintain skin integrity. A sufficient number of different positions is probably individual, but at least 6 different positions 3 cm apart are recommended.

## Data Availability

The authors will make the raw data supporting this article’s conclusions available upon request due to privacy.
